# Diseases of Women and Abdominal Surgery

**Published:** 1890-07

**Authors:** 


**Diseases of Women and Abdominal Surgery.** By LAWSON TAIT, F. R. C. S., Edin. and Eng., LL. D., M. D., Professor of Gynecology in Queen's College, Birmingham. Vol. I., 8vo, pp. viii.—543. Philadelphia: Lea Brothers & Co. 1889.

This book, like all that emanates from the pen of the distinguished author, will be read with interest by all who wish to keep themselves abreast of the literature of the day on the topics dealt with in its pages; and especially by those who have had an opportunity to partake of instruction at Mr. Tait's hands. It is probable that nothing that Mr. Tait has said in this work will change his status in the professional mind. He has already become established as authority on many questions, particularly those relating to the surgery of the abdomen. If we are to judge by results, certainly he must be given a foremost place in the history of that department of surgery. If, again, there are those who dissent from Mr. Tait's views on many questions, that need not — should not — prevent a fair judgment upon the successful work that he actually performs, nor detract from his established renown.

The work before us is characteristic of its author. It is direct in its statements, strong in its diction, sometimes polemic in its arguments, but always instructive in its lines. Whenever Mr. Tait criticises, which is not seldom, he does not fail to teach a lesson thereby. He does not seem to be critical for the mere sake of finding fault, but rather to enforce an argument. This point is too often overlooked by many critics, and Mr. Tait himself has not, in our view, been given sufficient credit in this direction. To find fault for the mere sake of gratifying a spirit of antagonism, is to be a bore; but to lay bare the weak points of a position with force and direction, even unto severity, becomes many times a necessity when an essential principle is involved that cannot otherwise be maintained. This last work of Mr. Tait will hardly add to or detract from his fame as an author or teacher, but it serves to group a mass of facts or opinions into a compact form for convenient reference, that have heretofore been scattered through journals and monographs. The general scope of the work is to discuss the wider range of gynecology, but its chief interest centers in the portion devoted to abdominal surgery, which, as might be expected, embraces the larger part of the treatise.

The subject of Ectopic Pregnancy and Pelvic Hematocele is taken up at page 438, and continues to the end of the book. We have heretofore (March, 1889,) devoted considerable space in our columns to an analysis of Mr. Tait's views on this subject, and we see no reason to change them or to repeat them now. We should be glad to supply that number of the JOURNAL to any who may desire it. It is a subject of such transcendent importance, in view of the frequency of its occurrence, and the mistakes in diagnosis that are happening, that nothing that will throw light upon it, or serve to enable the general practitioner to recognize the importance of seeking early and prompt aid in even suspected cases, should be overlooked.

The following, taken from a recent number of a newspaper published in Akron, O., is introduced here to show the medico-legal entanglements that a mistaken diagnosis may involve:

> A surgical operation was yesterday performed in this city with such remarkable results that it becomes necessary to make brief mention of it. For about a year Dr. I. J. Baughman, of South Akron, has been treating Miss Melissee Kintner, who resides on Bowery street with her sister, for ovarian tumor. It was thought that the tumor was caused by injuries which Miss Kintner received in an accident on the N. Y., P. &. O. Railroad, near Hudson, about eighteen months ago, and for which she obtained a judgment in court against the N. Y., P. & O. Company some time ago.
>
> It finally became evident, in the judgment of Dr. Baughman and other physicians, that the only chance to save the young woman's life was to operate upon her for the removal of the tumor. She consented to have the operation performed, and yesterday was the time set for it. Dr. H. S. Biggar, Professor of Surgery at the Cleveland Col'ege of Homeopathy, and Surgeon at the Huron-street Hospital, Cleveland, performed the operation, and besides Dr. Baughman, the physician in charge, there were present Drs. J. W. Rockwell, O. D. Childs, H. B. Carter, William Murdock, of this city, and H. W. Carter, of Cuyahoga Falls.
>
> To the profound astonishment of the operator, Prof. Biggar, and to his associate physicians, he discovered in performing the operation, not a tumor, but a child —a girl—weighing fully twelve pounds. The child was dead, and according to the judgment of the physicians present, had been dead for from one to three months. As stated above, the woman's trouble began over a year ago, and the case, on that account, and on account of the imperfect diagnosis, is one of the most remarkable in the medical annals of Summit county or Northern Ohio. The physicians were completely misled, and were profoundly astonished when the facts were revealed by the knife.

Miss Kitner is in a dangerous condition, and it is not expected that she will survive the operation.

We have published this clipping for the purpose of giving all the history of the case known to us. We assume, if this account is correct, that this was clearly a case of extra-uterine fetation, continuing to near term before the death of the fetus, and still the diagnosis was not made out until operation some months later revealed the true condition of affairs. Meanwhile a railway company is mulcted in damages for supposed injury. It would be interesting to examine the court proceedings at the trial to ascertain upon what testimony the verdict was allowed. Who were the expert witnesses, and what were the grounds on which their judgment was based? It would appear to us important that greater care should be exercised by the courts in admitting so-called expert testimony in obscure cases, to the end that none but men of acknowledged experience and judgment should be allowed to give *ex cathedra* opinions on the trial of medico-legal cases before judicial tribunals.

